# Impact of infertility duration on female sexual health

**DOI:** 10.1186/s12958-021-00837-7

**Published:** 2021-10-09

**Authors:** Meng Dong, Xiaoyan Xu, Yining Li, Yixian Wang, Zhuo Jin, Jichun Tan

**Affiliations:** 1 Center of Reproductive Medicine, Shengjing Hospital of China Medical University, 110072 Shenyang, China; 2 Key Laboratory of Reproductive Dysfunction Diseases and Fertility Remodelling of Liaoning Province, Shenyang, 110072 China; 3 School of Life Sciences, China Medical University, Shenyang, 110122 China

## Abstract

**Background:**

Infertility, an important source of stress, could affect sexual life. Extensive studies suggest that the incidence of sexual dysfunction is highly prevalent in infertile women. As the duration of infertility increases, the level of stress is also likely to increase even further, and this could aggravate psychological pain and cause sexual dysfunction. However, the effect of infertility duration on sexual health is unclear.

**Methods:**

We conducted a case-control study in which 715 patients participated between September 1,2020 and December 25, 2020. We included patients diagnosed with infertility (aged between 20 to 45), who were divided into four groups according to their infertility durations: ≤ 2 years (Group I, n = 262), > 2 years but ≤ 5 years (Group II, n = 282), > 5 years but ≤ 8 years (Group III, n = 97), and > 8 years (Group IV, n = 74). A questionnaire survey on female sexual functions and psychological depression was administered to participants, and their female sexual functions and depression status were measured using the Female Sexual Function Index (FSFI) and Patient Health Questionnaire (PHQ-9), respectively.

**Results:**

As the number of years of infertility increased, the PHQ-9 score as well as the incidence of psychological depression increased significantly (*p* < 0.05), but the total score of FSFI and those of its six domains/sub-scales were not significantly different among the four groups. An analysis of the relevant factors affecting sexual functions, using the multivariable logistic regression model, revealed that when the infertility duration was greater than 8 years, there was a significant increase in the incidence of sexual dysfunction [adjusted odds ratios (AOR) = 5.158, 95% confidence interval (CI): 1.935–13.746, *P* = 0.001], arousal disorder (AOR = 2.955, 95% CI: 1.194–7.314, *P* = 0.019), coital pain (AOR = 3.811, 95% CI: 1.045–13.897, *P* = 0.043), and lubrication disorder (AOR = 5.077, 95% CI: 1.340–19.244, *P* = 0.017).

**Conclusions:**

An increasing infertility duration is a risk factor for the occurrence of sexual dysfunction. Hence, as the infertility duration increases, the incidence of female sexual dysfunction and psychological distress could also increase, especially when the infertility duration is more than 8 years.

## Introduction

Sexual dysfunction is a global health problem, affecting approximately 41% of premenopausal women [1]. Female sexual dysfunction (FSD), defined as a sexual problem associated with personal distress [2], includes sexual desire disorder, arousal disorder, lubrication difficulties, orgasmic disorder, and pain disorders [3]. It results from the interaction of multiple physical, psychological, relational, and sociocultural factors [4]. In addition, factors, such as age, mental health, income and education levels, lifestyle, race, social background, gender, and endocrine disorders, could affect sexual functions [1, 2, 5].

Infertility is defined as having not been able to conceive after 12 months of unprotected sexual intercourse—specifically referring to penile penetration—in a heterosexual relationship [4], and the duration of the failure to conceive should be ≥ 12 months before an investigation is undertaken [6]. Extensive studies have reported that the diagnosis and treatment of infertility are related to the occurrence of sexual dysfunction [7–12]. As an important source of stress, infertility can affect the quality of sexual life [13]. Studies reveal that the incidence of sexual dysfunction is greater in infertile women than in those with normal fertility [14–17]. Issues such as negative effects of infertility treatment and pressure from family members cause tremendous stress on infertile patients, leading to both psychological and physical pain [12]. Our hypothesis is that as the duration of infertility increases, stress is likely to increase even further, and subsequently, it could aggravate the psychological pain and cause sexual dysfunction.

To the best of our knowledge, only one study has explored the impact of infertility duration on the occurrence of FSD [9]. In this study, 169 infertile women were divided into three groups according to their infertility duration: less than 2 years (Group I), 2–5 years (Group II), and 5 years and longer (Group III). This study found that as the infertility duration extended, the scores of all sexual domains decreased, except sexual satisfaction [9]. However, this study did not include potential confounding factors, and its sample size was relatively small. As far as we know, there is no high-quality study that compares the effects of infertility duration on sexual health. Therefore, our study uses a large sample to explore whether the infertility duration affects female sexual health.

## Materials and methods

This is a case-control study which was conducted at the Reproductive Medical Centre of Shengjing Hospital affiliated to China Medical University. A survey of female sexual function and psychological depression of patients with infertility was performed. A total of 715 infertile patients participated in the questionnaire survey between September 1, 2020 and December 25, 2020. Patients with infertility were grouped into four categories according to their infertility duration (defined as the time from these couples’ first attempt to conceive to their entering the study [4]): ≤ 2 years (Group I, n = 262), > 2 years but ≤ 5 years (Group II, n = 282), > 5 years but ≤ 8 years (Group III, n = 97), and > 8 years (Group IV, n = 74).

### Study participants

Inclusion criteria: This study’s participants included women who were diagnosed with infertility and younger than 45 years to prevent potential biases, such as age and endocrine environmental factors, given that the incidence of sexual dysfunction increases significantly in perimenopausal women due to a decline in their hormone levels [1, 18–23]. This criterion was consistent with the age in previous studies on the sexual functions of women with infertility [4, 9].

Exclusion criteria: Females diagnosed with 1) polycystic ovary syndrome (PCOS), 2) endometriosis, 3) premature ovarian failure, 4) diabetes, 5) high blood pressure, 6) lower genital tract abnormalities, 7) genitourinary infections, 8) genital prolapse, or 9) whose partners had severe male infertility, 10) whose partners had been diagnosed by specialists with sexual dysfunction, 11) presence of psychiatric conditions (that could cause sexual dysfunction), or 12) used drugs that affected sexual functions (e.g., selective serotonin reuptake inhibitors, as well as serotonin and norepinephrine reuptake inhibitors) [8]. We excluded several types of infertilities, such as endometriosis [24], PCOS [25, 26], premature ovarian failure [27, 28], and severe male infertility [29–31], that were reported to have affected sexual functions. Taking into account of previous studies, women with a total FSFI score < 8 were excluded because this score implies that they do not have sufficient sexual activity [4, 32, 33]. Participants who did not complete the questionnaire and whose answers were inconsistent (e.g. in questions that focused on sexual functions, “did not attempt intercourse” was selected in one question but not in others) were also excluded.

### Applied questionnaires (measurement)

The self-administered questionnaire comprised three parts. The first part was designed to collect information about the sociodemographic characteristics of patients with infertility: age, body mass index (BMI), primary/secondary infertility, smoking status (0=yes, 1=no), economic level, infertility duration, education level(0= ≤ high school, 1=college, 2=undergraduate, 3= ≥postgraduate), drinking alcohol status(1=usually, 2=sometimes, 3=rarely, 4=never), stress in work and life(0=very high, 1=high, 2=general, 3=low, 4=none), and frequency of physical exercise(0=none, 1= <1 time a week, 2=1 time a week, 3= ≥2 times a week).

The second part was a survey of participants' sexual health and sexuality for measuring the FSFI [34] and the four aspects of sexuality, that were adopted from a previous study’s questionnaire [24], including the questions on the importance of sex in general, dyspareunia, the possibility of orgasms in different sexual activities, and the duration of intercourse and foreplay. The importance of sex in general was assessed by asking the following multiple-choice questions: “Is sex important to you?” Response options were set as: very important, important, general, unimportant, and very unimportant. Dyspareunia was assessed by asking “Have you experienced difficulties during sexual intercourse?” Response options were set as: never, very rarely, rarely, sometimes, usually, and almost always. With regard to the possibility of reaching orgasm in different sexual activities, the following three questions were asked: 1. What is the possibility of your reaching orgasm through masturbating? 2. What is the possibility of your reaching orgasm during non-coital intercourse, such as foreplay or oral sex? 3. What is the possibility of your having an orgasm during sexual intercourse? The response options were set as: almost always, always, sometimes, rarely, never, and never tried. The duration of intercourse and foreplay were also evaluated by two questions, whose response options were <1, 1–2, 3–4, 5–7, 8–10, 11–15, 16–30, > 30 minutes and < 1, 2–10, 11–20, 21–30, 31–60, > 60 minutes, respectively. The FSFI includes 19 items and 6 domains of sexual functions (desire, arousal, lubrication, orgasm, satisfaction, and coital pain) based on the sexual status in the preceding 4 weeks [34]. The scores for each domain range from 1.2 to 6 or from 0 to 6, and the total score ranges from 2 to 36, with a Cronbach’s alpha value ≥ 0.82 [20]. The Chinese cut-off of the total FSFI score was ≤ 23.45, which indicated that the women could have sexual dysfunction [35, 36]. The cut-off scores for each domain were established as follows: ≤ 2.7 low desire, ≤ 3.15 arousal disorder, ≤ 4.05 lubrication disorder, ≤ 3.8 orgasm disorder, and ≤ 3.8 sexual pain [36].

The third part was a survey of psychological depression evaluated by Patient Health Questionnaire (PHQ-9) [37]. The items were scored on a 4-point scale from 0 to 3, with a total severity score ranging from 0 to 27. A cut-off score of ≥ 10 was used to assess the presence of depression with a Cronbach’s α value ranging from 0.73 to 0.95 [37].

The study’s participants comprised patients recruited from the Reproductive Medical Centre, who voluntarily participated and to whom anonymous and confidential questionnaires were distributed. They filled them out in a private setting (alone), without any time limit, and the completed questionnaires were placed in a box and collected altogether. The approval for this study was given by the Hospital’s Institutional Review Board for research on human subjects, and the participants were not reimbursed for their involvement.

### Statistical analyses

Data analysis was performed using the statistical software SPSS (version 22.0; SPSS Inc., Chicago, IL, USA). Categorical variables were summarised with counts(n) and percentages (%); continuous measures, with counts, means, standard deviations (SDs).

The chi-square test was used to compare the categorical variables and one-way analysis of variance (ANOVA) were used to compare numerical data. Multivariable logistic regression was employed to explore the factors that affect sexual function and each of the five outcomes of desire disorder, arousal disorder, lubrication disorder, orgasm disorder, and coital pain disorder. *P* value, *OR*, and 95% CI were evaluated, and a two-tailed *p* value < 0.05 indicated statistical significance.

## Results

### Participants’ demographic characteristics

A total of 791 patients participated in this study, and 76 patients were excluded from the study (52 patients failed to complete all the questions, 23 provided inconsistent answers, and 1 had a total FSFI score below 8). Thus, responses of 715 participants were included in the analysis and the response rate was 90.39%. Table 1 summarizes the participants’ demographic characteristics. Of the participants, the average age was 32.68 years (SD, 4.37; range 21-44) and the average infertility duration was 4.08 years (SD, 3.05; range 1-20). There are significant differences in age, BMI, education levels, and physical exercise frequency among the four groups of participants (*p* < 0.05). There were no significant differences in primary or secondary infertility stress, income levels, or smoking and drinking status among the four groups of participants (*p* > 0.05) (Table 1).

**Table 1:** Demographic characteristics of the study participants (n = 715)

Characteristics	Group I (n = 262)	Group II (n = 282)	Group III (n=97)	Group IV (n=74)	*P*- value
	Mean ±SD / n (%)	Mean ±SD / n (%)	Mean ±SD / n (%)	Mean ±SD / n (%)	
Age (years)	31.13 ± 4.36	32.35 ± 3.84	34.25 ± 3.35	37.41 ± 3.59	0.000**
BMI (kg/m^2^)	23.06 ± 3.27	23.45 ± 3.61	23.49 ± 3.11	25.14 ± 3.04	0.000**
Primary infertility	160 (61.07)	177 (62.77)	58 (59.79)	44 (59.46)	0.683
Secondary infertility	102 (38.93)	105 (37.23)	39 (40.21)	30 (40.54)	
Smoking status:					0.555
Smoker	13 (5.0)	16 (5.7)	2 (2.1)	4 (5.4)	
Non-smoker	249 (95.0)	266 (94.3)	95 (97.9)	70 (94.6)	
Annual income (ten thousand yuan)					0.782
< 5	127 (48.5)	147 (52.1)	53 (54.6)	38 (51.4)	
5-10	80 (30.5)	69 (24.5)	30 (30.9)	24 (32.4)	
10-15	30 (11.5)	35 (12.4)	7 (7.2)	5 (6.8)	
15-20	13 (5.0)	13 (4.6)	3 (3.1)	3 (4.1)	
> 20	12 (4.6)	18 (6.4)	4 (4.1)	4 (5.4)	
Education					0.000**
≤ High school	72 (27.5)	93 (33.0)	36 (37.1)	45 (60.8)	
College	78 (29.8)	73 (25.9)	20 (20.6)	9 (12.2)	
Undergraduate	83 (31.7)	97 (34.4)	37 (38.1)	17 (23.0)	
≥ Postgraduate	29 (11.1)	19 (6.7)	4 (4.1)	3 (4.1)	
Stress in work and life					0.945
Very high	16 (6.1)	15 (5.3)	3 (3.1)	3 (4.1)	
High	48 (18.3)	59 (20.9)	18 (18.6)	15 (20.3)	
General	144 (55.0)	146 (51.8)	53 (54.6)	43 (58.1)	
Low	37 (14.1)	49 (17.4)	16 (16.5)	9 (12.2)	
None	17 (6.5)	13 (4.6)	7 (7.2)	4 (5.4)	
Physical exercise frequency					0.003**
None	99 (37.8)	91 (32.3)	27 (27.8)	24 (32.4)	
< 1 time a week	94 (35.9)	94 (33.3)	24 (24.7)	26 (35.1)	
1 time a week	44 (16.8)	40 (14.2)	18 (18.6)	10 (13.5)	
≥ 2 times a week	25 (9.5)	57 (20.2)	28 (28.9)	14 (18.9)	
Drinking alcohol					0.451
Usually	2 (0.8)	1 (0.4)	1 (1.0)	0 (0)	
Sometimes	22 (8.4)	16 (5.7)	7 (7.2)	5 (6.8)	
Rarely	106 (40.5)	88 (31.2)	32 (33.0)	29 (39.2)	
Never	132 (50.4)	177 (62.8)	57 (58.8)	40 (54.1)	

Data was described as mean ± SD or n (%)

*Abbreviations*: *SD* standard deviation, *BMI* body mass index

* *p* < 0.05, ** *p* < 0.01

### Comparison of female psychological and sexual health among patients with different infertility duration

As the number of years of infertility increased, the PHQ-9 score and the incidence of psychological depression increased significantly (*p* < 0.05). The total FSFI score and those of its six domains were not significantly different among the four groups. Likewise, the incidence of sexual dysfunction, low desire, arousal disorder, lubrication disorder, orgasm disorder, and sexual pain was not significantly different among the four groups (*p* > 0.05) (Table 2).

**Table 2:** Female psychological and sexual health among patients with different infertility duration

	Group I (n = 262)	Group II (n = 282)	Group III (n=97)	Group IV (n=74)	*P*-value
	Mean ±SD / n (%)	Mean ±SD / n (%)	Mean ±SD / n (%)	Mean ±SD / n (%)	
PHQ -9 score	7.01 ± 3.03	7.39 ± 2.98	8.46 ± 2.90	9.39 ± 2.95	0.000**
Incidence of depression	31 (11.8)	49 (17.4)	27 (27.8)	31 (41.9)	0.000**
Sexual life frequency (per month)	5.05 ± 2.88	4.87 ± 2.92	4.66 ± 2.98	4.75 ± 3.25	0.685
FSFI score	27.03 ± 3.86	26.64 ± 3.77	26.48 ± 4.42	26.63 ± 3.59	0.553
Incidence of sexual dysfunction	45 (17.2)	45 (16.0)	20 (20.6)	12 (16.2)	0.765
Sexual desire score	3.46 ± 0.77	3.33 ± 0.69	3.30 ± 0.75	3.24 ± 0.89	0.052
Incidence of low desire	45 (17.2)	58 (20.6)	21 (21.6)	17 (23.0)	0.589
Arousal ability score	4.12 ± 0.96	4.00 ± 0.90	3.93 ± 1.03	3.90 ± 0.92	0.158
Incidence of arousal disorder	41 (15.6)	40 (14.2)	19 (19.6)	13 (17.6)	0.620
Vaginal lubricity score	5.16 ± 0.76	5.14 ± 0.83	5.15 ± 0.86	5.13 ± 0.72	0.995
Incidence of lubrication disorder	25 (9.5)	24 (8.5)	8 (8.2)	5 (6.8)	0.891
Orgasm score	4.60 ± 0.97	4.55 ± 0.96	4.57 ± 0.98	4.55 ± 0.90	0.933
Incidence of orgasm disorder	54 (20.6)	46 (16.3)	22 (22.7)	14 (18.9)	0.454
Satisfaction score	4.73 ± 0.91	4.64 ± 0.94	4.59 ± 1.06	4.60 ± 0.83	0.504
Coital pain score	4.96 ± 0.89	4.97 ± 0.88	4.95 ± 0.97	5.22 ± 0.72	0.143
Incidence of coital pain	28 (10.7)	27 (9.6)	11 (11.3)	4 (5.4)	0.546
Dyspareunia					0.092
Almost always	0 (0.0)	1 (0.4)	2 (2.1)	1 (1.4)	
Usually	1 (0.4)	7 (2.5)	3 (3.1)	0 (0.0)	
Sometimes	48 (18.3)	46 (16.3)	13 (13.4)	10 (13.5)	
Rarely	66 (25.2)	64 (22.7)	24 (24.7)	13 (17.6)	
Very rarely	47 (17.9)	56 (19.9)	23 (23.7)	11 (14.9)	
Never	100 (38.2)	108 (38.3)	32 (33.0)	39 (52.7)	

*PHQ-9* Patient Health Questionnaire, *FSFI* Female Sexual Function Index

No significant differences were identified in the frequency of orgasms during different sexual activities and sexual intercourse or foreplay duration in patients with different infertility durations among the four groups (*p* > 0.05) (Table 3).

**Table 3:** Importance of sex life, duration of intercourse and foreplay, and frequency of orgasm during different sexual activities

Characteristics	Group I (n = 262)	Group II (n = 282)	Group III (n=97)	Group IV (n=74)	*P*- value
	n (%)	n (%)	n (%)	n (%)	
Importance of sex life					0.346
Very important	12 (4.6)	6 (2.1)	5 (5.2)	1 (1.4)	
Important	111 (42.4)	97 (34.4)	35 (36.1)	29 (39.2)	
Neutral	127 (48.5)	167 (59.2)	50 (51.5)	40 (54.1)	
Unimportant	11 (4.2)	10 (3.5)	7 (7.2)	4 (5.4)	
Very unimportant	1 (0.4)	2 (0.7)	0 (0.0)	0 (0.0)	
Duration of intercourse					0.119
<1min	0 (0.0)	1 (0.4)	1 (1.0)	0 (0.0)	
1-2 min	6 (2.3)	7 (2.5)	1 (1.0)	1 (1.4)	
3-4 min	30 (11.5)	31 (11.0)	13 (13.4)	14 (18.9)	
5-7 min	40 (15.3)	78 (27.7)	21 (21.6)	20 (27.0)	
8-10 min	70 (26.7)	64 (22.7)	29 (29.9)	17 (23.0)	
11-15 min	57 (21.8)	44 (15.6)	13 (13.4)	11 (14.9)	
16-30 min	46 (17.6)	50 (17.7)	17 (17.5)	8 (10.8)	
> 30 min	13 (5.0)	7 (2.5)	2 (2.1)	3 (4.1)	
Duration of foreplay					0.595
<1min	37 (14.1)	47 (16.7)	15 (15.5)	9 (12.2)	
2-10 min	181 (69.1)	174 (61.7)	63 (64.9)	52 (70.3)	
11-20 min	37 (14.1)	52 (18.4)	15 (15.5)	12 (16.2)	
21-30 min	7 (2.7)	6 (2.1)	4 (4.1)	1 (1.4)	
31-60 min	0 (0.0)	3 (1.1)	0 (0.0)	0 (0.0)	
Frequency of orgasm during different sexual activities					
Masturbating					0.720
Almost always	36 (13.7)	29 (10.3)	6 (6.2)	6 (8.1)	
Usually	26 (9.9)	30 (10.6)	10 (10.3)	8 (10.8)	
Sometimes	46 (17.6)	52 (18.4)	18 (18.6)	15 (20.3)	
Rarely	36 (13.7)	38 (13.5)	22 (22.7)	12 (16.2)	
Never	45 (17.2)	56 (19.9)	13 (13.4)	12 (16.2)	
Never tried	73 (27.9)	77 (27.3)	28 (28.9)	21 (28.4))	
Non-coital activities					0.713
Almost always	15 (5.7)	11 (3.9)	3 (3.1)	3 (4.1)	
Usually	26 (9.9)	26 (9.2)	5 (5.2)	7 (9.5)	
Sometimes	72 (27.5)	71 (25.2)	24 (24.7)	16 (21.6)	
Rarely	57 (21.8)	59 (20.9)	24 (24.7)	18 (24.3)	
Never	43 (16.4)	45 (16.0)	14 (14.4)	17 (23.0)	
Never tried	49 (18.7)	70 (24.8)	27 (27.8)	13 (17.6)	
Sexual intercourse					0.515
Almost always	13 (5.0)	10 (3.5)	2 (2.1)	4 (5.4)	
Usually	76 (29.0)	74 (26.2)	27 (27.8)	15 (20.3)	
Sometimes	104 (39.7)	138 (48.9)	40 (41.2)	37 (50.0)	
Rarely	58 (22.1)	47 (16.7)	24 (24.7)	14 (18.9)	
Never	10 (3.8)	13 (4.6)	3 (3.1)	4 (5.4)	
Never tried	1 (0.4)	0 (0.0)	1 (1.0)	0 (0.0)	

### Multivariable risk factors affecting sexual functions

A multivariable logistic regression model was used to analyze relevant factors— age; BMI; income, education, and stress levels; frequency of sex and physical exercise; smoking and drinking status; and psychological distress—that affect the sexual function.

The results reveal that as the infertility duration increases, the incidence of sexual dysfunction also increases. When infertility duration was greater than 8 years, the values were as follows: low desire (AOR = 1.252, 95% CI: 0.590-2.656, *P* = 0.558), arousal disorder (AOR = 2.955, 95% CI: 1.194-7.314, *P* = 0.019), orgasmic disorder (AOR = 2.089, 95% CI: 0.876-4.984, *P* = 0.097), coital pain (AOR = 3.811, 95% CI: 1.045-13.897, *P* = 0.043), and lubrication disorder (AOR = 5.077, 95% CI: 1.340-19.244, *P* = 0.017), and the incidence of sexual dysfunction (AOR = 5.158, 95% CI: 1.935-13.746, *P* = 0.001). Among them, the incidence of arousal disorder, coital pain, lubrication disorder, and sexual dysfunction significantly increased (*p* < 0.05). The state of depression was a risk factor for arousal disorder, orgasmic disorder, coital pain, lubrication disorder, and the incidence of sexual dysfunction, but not for sexual desire. Factors significantly and independently associated with low desire were lower frequency of sex and lower education levels. A lower likelihood of arousal disorder was significantly associated with infertility duration of greater than 8 years, increased age, smoking status, and high levels of psychological distress. Orgasmic disorder was significantly associated with BMI and psychological distress. A higher coital pain was significantly associated with infertility duration of greater than 8 years, higher frequency of alcohol consumption, and psychological distress. The risk factors for lubrication disorder were infertility duration of greater than 8 years, lower frequency of sexual life, high stress levels, and psychological distress. A higher likelihood of sexual dysfunction was observed in patients with increasing infertility duration, increasing age, lower frequency of sexual life, and high levels of psychological distress (Table 4).

**Table 4:** Factors associated with sexual dysfunction, multivariable-adjusted odds ratios and 95% confidence intervals

Independent variable	Low desire	Arousal disorder	Orgasmic disorder	Coital pain	Lubrication disorder	Sexual dysfunction
Infertility duration						
≤ 2 years	1.000	1.000	1.000	1.000	1.000	1.000
2-5 years	1.299 (0.594-2.843) P=0.512	2.187 (0.884-5.412) P=0.090	1.624 (0.682-3.872) P=0.274	2.972 (0.810-10.903) P=0.100	2.067 (0.540-7.916) P=0.289	2.995 (1.137-7.891) P=0.026*
5-8 years	1.410 (0.698-2.851) P=0.338	2.029 (0.858-4.798) P=0.107	1.318 (0.574-3.026) P=0.514	3.198 (0.924-11.065) P=0.066	3.365 (0.950-11.917) P=0.060	3.553 (1.410-8.955) P=0.007**
> 8 years	1.252 (0.590-2.656) P=0.558	2.955 (1.194-7.314) P=0.019*	2.089 (0.876-4.984) P=0.097	3.811 (1.045-13.897) P=0.043*	5.077 (1.340-19.244) P=0.017*	5.158 (1.935-13.746) P=0.001**
Age (years)	1.048 (0.997-1.102) P=0.065	1.096 (1.033-1.162) P=0.002**	1.013 (0.958-1.072) P=0.643	1.005 (0.936-1.080) P=0.886	1.034 (0.957-1.117) P=0.399	1.093 (1.029-1.161) P=0.004**
Body mass index	1.009 (0.953-1.068) P=0.753	0.959 (0.894-1.028) P=0.241	0.927 (0.868-0.991) P=0.025*	1.029 (0.951-1.115) P=0.474	1.004 (0.918-1.098) P=0.932	1.019 (0.952-1.092) P=0.582
Income level	1.019 (0.857-1.212) P=0.828	0.937 (0.762-1.154) P=0.542	1.031 (0.855-1.244) P=0.746	0.903 (0.704-1.158) P=0.423	0.823 (0.619-1.096) P=0.182	0.852 (0.683-1.061) P=0.153
Sexual life frequency (per month)	0.890 (0.822-0.963) P=0.004**	0.911 (0.829-1.000) P=0.051	0.938 (0.863-1.020) P=0.134	0.946 (0.850-1.052) P=0.304	0.791(0.688-0.909) P=0.001**	0.888 (0.805-0.981) P=0.019*
Education level	0.780 (0.629-0.967) P=0.024*	0.993 (0.779-1.265) P=0.952	1.121 (0.894-1.405) P=0.322	1.213 (0.906-1.624) P=0.195	0.969 (0.704-1.333) P=0.847	1.193 (0.934-1.523) P=0.158
Stress level	1.021 (0.819-1.273) P=0.853	0.935 (0.729-1.198) P=0.595	0.890 (0.700-1.131) P=0.340	1.004 (0.740-1.362) P=0.981	0.700 (0.501-0.977) P=0.036*	0.774 (0.596-1.005) P=0.054
Physical exercise frequency	0.839 (0.696-1.011) P=0.066	0.885 (0.715-1.095) P=0.260	0.892 (0.730-1.090) P=0.264	0.960 (0.744-1.237) P=0.750	1.146 (0.871-1.508) P=0.329	0.870 (0.701-1.081) P=0.209
Smoking status	0.654 (0.297-1.442) P=0.293	0.406 (0.168-0.980) P=0.045*	1.410 (0.474-4.196) P=0.537	3.210 (0.613-16.808) P=0.167	1.823 (0.421-7.890) P=0.422	0.728 (0.267-1.984) P=0.535
Drinking status	1.082 (0.803-1.459) P=0.604	1.283 (0.899-1.829) P=0.169	0.957 (0.692-1.325) P=0.792	0.597 (0.407-0.876) P=0.008**	0.834 (0.541-1.283) P=0.408	1.056 (0.741-1.504) P=0.765
Psychological distress	1.059 (0.996-1.126) P=0.065	1.317 (1.224-1.416) P=0.000**	1.351 (1.252-1.458) P=0.000**	1.246 (1.157-1.342) P=0.000**	1.320 (1.219-1.429) P=0.000**	1.464 (1.344-1.596) P=0.000**

* *p* < 0.05, ** *p* < 0.01

## Discussion

In this large-scale case-control study, we have reported the impact of infertility duration on the FSD of patients with infertility and analyzed the risk factors that affect their sexual health. The multivariate logistic regression model revealed that an increasing infertility duration is a risk factor for the occurrence of sexual dysfunction.

According to our literature review, only one clinical study [9] investigated the impact of infertility duration on FSD. It found that sexual dysfunction, including low desire, arousal disorder, orgasm disorder, coital pain, and lubrication disorder were significantly higher in the > 5 years group, as compared with the < 2 years and 2–5 years infertility duration groups [9]. However, this study did not include interferences of potential confounding factors that would affect sexual functions, such as age, BMI, income level, education level, smoking, and drinking status. Moreover, it did not indicate the exclusion criteria, and its sample size was relatively small. Thus, we believe the conclusions drawn from this study have substantial limitations.

In another study on the impact of infertility-related distress on female sexual functions, infertility duration as a variable of infertility-related factors and hierarchical binary logistic regression showed that infertility-related factors (infertility duration) were insignificantly associated with sexual functions [4]. The study also compared the infertility duration of infertile patients with and without sexual dysfunction, which were 5.4 ± 3.4 versus 5.9 ± 3.9, respectively, and found no significant difference [4]. Its research purpose, inclusion and exclusion criteria, and method were all different from ours, thus possibly explaining the difference in results.

Another study conducted in Turkey (including 352 infertile and 301 normal fertility women) aimed to evaluate the prevalence and risk factors for sexual dysfunction in females with infertility. The results of multivariate logistic regression showed that a > 3 years duration of both marriage and infertility, together with a history of previous infertility treatment were risk factors for sexual dysfunction [7]. This study’s conclusion is partially consistent with ours, but it did not further divide the infertility duration. In our study, we identified that an infertility duration > 8 years was a risk factor for arousal, pain, lubrication disorder, and sexual dysfunction, but not for desire and orgasmic dysfunction.

Infertile patients undergoing assisted reproduction often report sexual disorders, especially in terms of decreased interest and desire for sex, poorer arousal and lubrication, and orgasm difficulties [12, 38, 39]. A study found that sexual desire disorder occurs differently in women of different ages, and the prevalence of this diagnosis is reported at 8.9% in 18–44-year-olds, 12.3% in 45–64-year-olds, and 7.4% in the age group > 65 years [40]. In our study, the loss of sexual desire increased as the years of infertility increased (an average of 17.2% increased to 23.0%), but there was no significant difference. The high incidence rate of low sexual desire was probably owing to the following reasons: when women with infertility realize that normal sexual intercourse does not lead to pregnancy, they may lose their desire for sex. Moreover, sexual intercourse for the sake of childbirth, rather than simply for pleasure, may also inhibit the occurrence of sexual desire.

This study also found that an infertility duration of greater than 8 years is a risk factor for sexual arousal disorder, that is also related to age, depression, and smoking in our study. Previous research found that unhappy life events, psychosocial distress, drug use, gynecological disorders, and disruptions to hormone production could precipitate sexual arousal disorder [18]. Other studies also believe that smoking increases the incidence of sexual dysfunction [41–43].

A few studies have reported the possibility of infertile patients achieving orgasms during different sexual activities, which may be closely related to sexual satisfaction [24]. However, our study did not identify differences in sexual satisfaction among patients with different infertility durations, or find possible differences in their orgasms during different sexual activities. Therefore, we believe that the infertility duration did not have a significant effect on the orgasm function. Conversely, we noted that during different sexual activities, the possibility of patients with infertility achieving orgasms always or often is less than 30%, which is much lower than that reported for those with normal fertility [24]. This may be because sexual intercourse is purposeful (fertility) rather than out of instinct and pleasure.

The duration of sexual intercourse mainly affects the possibility of orgasm dysfunction. Researchers believe that the likelihood or consistency of partnered orgasm among women is associated with the duration of penile-vaginal intercourse, but not with that of foreplay [44]. An extremely short foreplay duration could indicate that the partner has insufficient emotional intimacy during sex, whereas an extremely long foreplay duration could indicate intercourse difficulties, which could also affect sexual satisfaction. In our group, we did not identify any difference between either. Hence, it can be seen that the infertility duration has a minimal effect on orgasm function or sexual satisfaction.

In this study, we observed that the PHQ-9 score increases significantly with the increase in infertility duration. When we conducted multivariate regression analysis with depressive status as a potential confounding factor, we observed that depression was a risk factor for sexual dysfunction, except for sexual desire. Therefore, it can be speculated that the increase in the infertility duration increases the incidence of sexual dysfunction by affecting these women psychologically and emotionally. Many studies have reported the impact of psychological depression on sexual functions in patients with infertility [4, 12, 45]. With the increase of infertility duration, infertility-related stress also gradually increases, which in turn affects the mental health of infertile patients [46]. Research comparisons have found that infertility for more than 3 years significantly increases anxiety and depressed moods, and decreases the quality of life of patients with infertility [46]. Decreased sexual function will seriously affect the quality of life and damage mental health. Conversely, a depressed mental state could also increase the incidence of sexual dysfunction [7].

The cause of FSD is complex and multifactorial. A new study reviews its common etiologies and risk factors—age and menopausal status, psychiatric conditions (anxiety and depression), medical conditions (diabetes, hypertension, neurologic disease, premature ovarian failure, and gynecologic diseases), stress (emotional or environmental), medications (psychotropic medications, antihypertensives, histamine blockers, and hormonal medications), relationship and lifestyle—that may play a role [2]. To increase the reliability of our study’s results, some above-mentioned causes and risk factors were excluded. Furthermore, factors such as stress (emotional or environmental), relationships, and lifestyle that were difficult to evaluate were not discussed in our study, and hence, not included in our exclusion criteria, which could be a limitation.

This study also has some other limitations. First, limitations arise from its strict exclusion criteria. Second, we made assumptions relating to heterosexuality, given that sexual orientation information was not collected from the participants. Moreover, because ours was a retrospective study, a large sample of multi-center experiments is required to further confirm its conclusions.

## Conclusions

As the infertility duration increases, the incidence of FSD and psychological distress may increase, especially when the infertility duration is more than 8 years. When treating infertility, especially in patients who have been infertile for a long time, reproductive doctors should also pay attention to the evaluation of their sexual health. The psychological stress caused by prolonged infertility may lead to the occurrence of sexual dysfunction, which in turn, further increases psychological distress. Attention should be paid to such patients’ sexual and mental health, so that reproductive doctors can better evaluate and manage infertile women’s sexual problems.

## Data Availability

The datasets used and/or analyzed during the current study are available from the first author on reasonable request.
